# Gene Expression Analysis of *In Vivo* Fluorescent Cells

**DOI:** 10.1371/journal.pone.0001151

**Published:** 2007-11-07

**Authors:** Konstantin Khodosevich, Dragos Inta, Peter H. Seeburg, Hannah Monyer

**Affiliations:** 1 Department of Clinical Neurobiology, Interdisciplinary Center for Neuroscience, University of Heidelberg, Heidelberg, Germany; 2 Department of Molecular Neuroscience, Max-Planck-Institute for Medical Research, Heidelberg, Germany; Institut Pasteur Korea, Republic of Korea

## Abstract

**Background:**

The analysis of gene expression for tissue homogenates is of limited value because of the considerable cell heterogeneity in tissues. However, several methods are available to isolate a cell type of interest from a complex tissue, the most reliable one being Laser Microdissection (LMD). Cells may be distinguished by their morphology or by specific antigens, but the obligatory staining often results in RNA degradation. Alternatively, particular cell types can be detected *in vivo* by expression of fluorescent proteins from cell type-specific promoters.

**Methodology/Principal Findings:**

We developed a technique for fixing *in vivo* fluorescence in brain cells and isolating them by LMD followed by an optimized RNA isolation procedure. RNA isolated from these cells was of equal quality as from unfixed frozen tissue, with clear 28S and 18S rRNA bands of a mass ratio of ∼2∶1. We confirmed the specificity of the amplified RNA from the microdissected fluorescent cells as well as its usefulness and reproducibility for microarray hybridization and quantitative real-time PCR (qRT-PCR).

**Conclusions/Significance:**

Our technique guarantees the isolation of sufficient high quality RNA obtained from specific cell populations of the brain expressing soluble fluorescent marker, which is a critical prerequisite for subsequent gene expression studies by microarray analysis or qRT-PCR.

## Introduction

There is increasing interest in tissue or cell type-specific gene expression analysis to identify genes involved in diseases, cell fate determination, or response to external stimuli. Researchers have attempted to develop methods for the isolation of homogeneous cell populations, such as flow cytometry and mechanical dissection, but these methods have practical limitations. Laser Microdissection (LMD), developed a decade ago [1] and currently used routinely in clinical and basic research applications, has permitted the isolation of distinct cell populations from complex tissues. It allows for precise analysis of DNA, RNA or proteins from cells of interest. Cells may be conveniently distinguished on the basis of morphology or specific antigens, which is achieved by staining with different dyes or specific antibodies. Incubation with these agents does not interfere with the integrity of DNA or protein isolated from microdissected cells, but compromises RNA quality due to RNase activity [2], [3]. In spite of the existence of staining protocols optimized for RNA quality[4], [5], RNA degradation that occurs during staining procedures is still an unresolved problem [6], [7]. Furthermore, only few cell types can be identified by cell morphology and specific antibodies for immunohistochemistry are available only for a limited range of proteins.

More recently, the identification of distinct cell types has been facilitated by the transgenic expression of fluorescent proteins. A major advance has been the generation of numerous transgenic mouse lines that express EGFP (enhanced green fluorescent protein) in defined cell populations [8], [9]. Also, to date, several techniques for *in vivo* fluorescent labeling of particular cell types/populations have been developed including viral or naked DNA delivery [10], [11]. However, there is a clear need for a reliable technique to separate specific *in vivo* fluorescent neural cell populations for subsequent gene expression analysis. We have now developed such a technique for the harvesting by LMD of fluorescent cells from brain tissue with subsequent RNA isolation and gene expression analysis by real-time PCR and microarrays. We demonstrate high quality of the isolated RNA from a defined cell population, the periglomerular cells of the olfactory bulb, and show the usefulness and specificity of the technique for subsequent gene expression studies by quantitative real-time PCR and microarrays studies.

## Results

We employed in our study transgenic 5HT3_A_-EGFP mice, in which the enhanced green fluorescent protein, EGFP, is expressed from the promoter of the serotonin receptor gene *5HT3_A_*. The unique and faithful expression pattern of the transgene has been reported elsewhere (Inta et al., manuscript submitted). During early postnatal life, EGFP is specifically expressed in neuroblasts migrating from the subventricular zone (SVZ) prominently to the olfactory bulb where neuroblasts mature into granule and periglomerular cells, subpopulations of GABAergic interneurons. Green fluorescent cells are clearly distinguishable in the olfactory bulb as well as on their route to the olfactory bulb from the SVZ.

To fix the fluorescence in the cells of interest, we perfused the mice with paraformaldehyde (PFA, 0.5% or 2%) and then cryoprotected the fixed tissue by sucrose to preserve tissue morphology. Perfusion is a fast and effective way for complete fixation of tissues, which is very important when working with RNA, as it prevents RNase activity.

**Figure 1:**
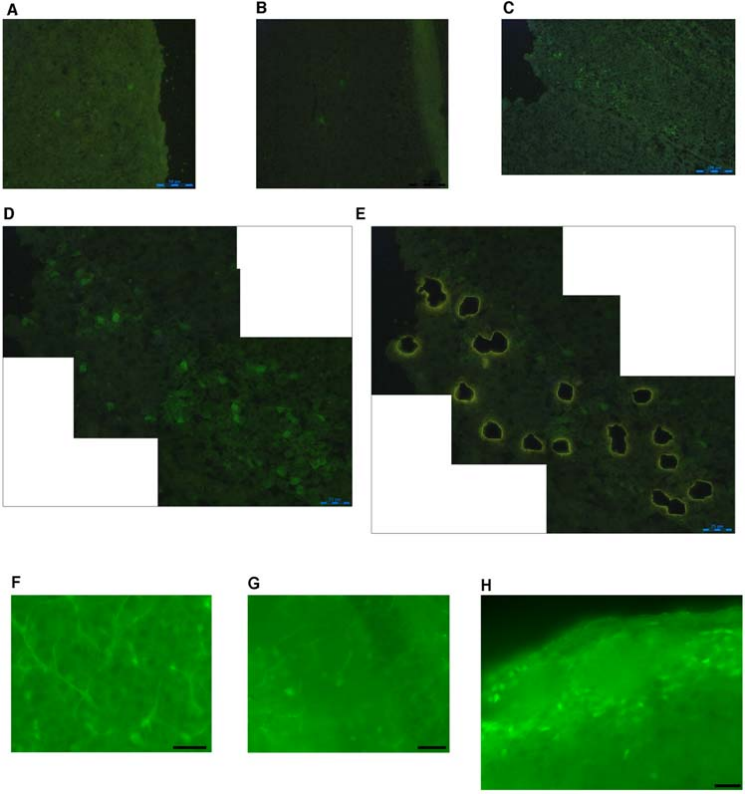
Green fluorescent cells in the 5HT3_A_-EGFP mouse brain. Cortex (*A*) and (*F*), hippocampus (*B*) and (*G*), and olfactory bulb (*C*) and (*H*) fixed by LMD-adapted and standard fixation protocol, respectively. (*D*) and (*E*) Periglomerular cell layer of olfactory bulb before and after microdissection, respectively. (*A*)–(*E*) images were made on a LMD microscope with brain sections mounted on membrane polyester slides. (*F*)–(*H*) images were made on an upright fluorescent microscope. For images (*A*)–(*E*) LMD microscope-generated scale bars are shown in the left down corner, (*A*)-50 µm, (*B*) and (*C*)-100 µm, (*D*) and (*E*)-25 µm. For images (*F*)–(*H*) scale bar is 25 µm.

Fixed brains were frozen, and sliced into 6–8 µm sagittal sections. Although PFA fixation alone was sufficient to preserve fluorescence for a short time, it was not enough to keep intensity of fluorescence signal longer then 30–60 min (data not shown). Therefore, sections were additionally fixed and dehydrated by alcohol, following clearing by xylene. Incubation in a precipitative fixative, such as alcohol, constitutes an additional fixation step, necessary not only to remove the water to prevent RNA degradation, but also to render the aldehyde-crosslinks more stable, thus preserving the fluorescence. Alcohol fixation alone also was not sufficient to preserve fluorescence of the soluble EGFP and prevent it from leaching out and diffusing to neighboring tissue making it impossible to specifically identify green fluorescent cells (data not shown). To identify the conditions that best preserve tissue morphology and cause minimal tissue autofluorescence, we tested different alcohols. Thus, 50% ethanol followed by 100% n-butanol was found to be optimal. Different brain areas containing green fluorescent cells are shown in Fig. 1A–C (for comparison with standard fixation procedure by 4% PFA in fluorescence preservation, see the same brain areas in Fig. 1F–H). The concentration of PFA was not found to influence specific fluorescence-there was no notable difference when 0.5% or 2% PFA was used-but autofluorescence was reduced at lower PFA concentrations.

Sections containing the periglomerular cell layer of olfactory bulb were mounted directly on membrane slides for LMD and immediately processed by alcohol dehydration/fixation. Leaving mounted sections even at −70°C was found to impair tissue morphology. Since membrane slides are not as solid as glass slides, and mounted tissue might lead to membrane shrinkage. Green fluorescent periglomerular cells were microdissected into dry 0.2 ml tube caps coated with silicon (Fig. 1D, E). To minimize RNA degradation we did not use any collecting liquid. After dissecting 3,000–5,000 cells, we isolated their RNA by a modified proteinase K/acid phenol method.

**Figure 2:**
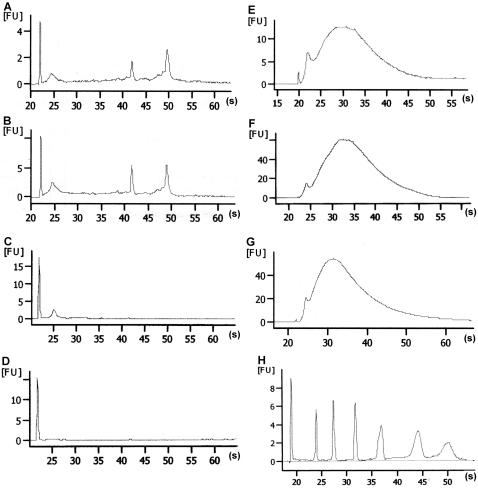
RNA analysis by the Bioanalyzer 2100. (*A*) RNA isolated from fixed tissue by the optimized proteinase K/acid phenol method described here. (*B*) RNA isolated from frozen tissue by the optimized proteinase K/acid phenol method. (*C*) RNA isolated from fixed tissue by TRIzol method. (*D*) RNA isolated from fixed tissue by RNeasy Micro Kit. *(E)* One round of amplification of RNA isolated from fixed tissue by the optimized proteinase K/acid phenol method. (*F*) One round of amplification of Ambion Control RNA. (*G*) Two rounds of amplification of RNA isolated from fixed tissue by the optimized proteinase K/acid phenol method. (*H*) RNA ladder: first peak is RNA marker, next mark 200, 500, 1000, 2000, 4000 and 6000 nt.

As methylene bridges formed by aldehyde fixation are reversible [12], we were able to reverse the extensive network of aldehyde crosslinking by an optimized proteinase K/SDS lysis solution and a subsequent treatment with phenol pH 4.2. Using this RNA isolation procedure, we obtained from the fixed tissue RNA of the same quality as that of RNA isolated from unfixed frozen tissue (Fig. 2A, B, respectively). The 28S∶18S rRNA ratio in the samples obtained was around 2∶1, which is excellent for RNA isolated from tissues by LMD [13]. Furthermore, the yield of RNA from the fixed tissue (6–9 ng from 3,000–5,000 cells) exceeded that from frozen tissue (4–7 ng from 3,000–5,000 cells), probably due to inactivation of RNases by aldehyde fixation.

We compared our procedure for RNA isolation with the standard TRIzol (Invitrogen, Germany) method and the RNeasy Micro Kit (QIAGEN, Germany). Yield and quality of RNA obtained by these methods were inferior to those obtained by our procedure (Fig. 2C, D), most likely due to the lack of reversing the network of aldehyde crosslinks.

We next amplified 2–3 ng of periglomerular cell RNA isolated by the method described above using a MessageAmp II aRNA Amplification Kit (Ambion, USA). The yield and size range of the amplified RNA were comparable to those obtained for the Ambion Control RNA (Ambion, USA) (Fig. 2E, F, respectively). The average length of the amplified mRNA pool (typical yield ∼50 ng) was around 1,000 nucleotides and was comparable to the Ambion RNA control. The length of the mRNA was judged by comparing it with the Ambion RNA Marker (Fig. 2H). After a second round of RNA amplification we typically obtained 50–100 µg of RNA of a 200–2,000 nucleotide range (Fig. 2G). Thus, we were able to obtain from 3,000–5,000 microdissected fluorescent cells high quality RNA that suffices for several microarray hybridizations.

**Figure 3:**
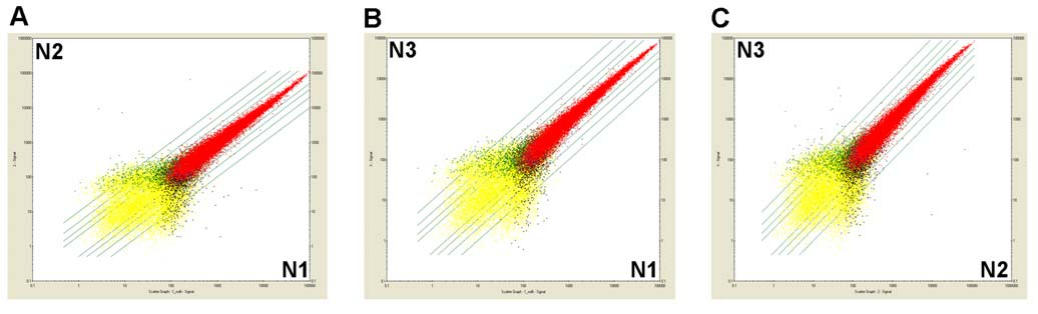
Scatter plots of signal detection values obtained in three independent microarray hybridizations. (*A*) Scatter plot between array N1 and array N2. (*B*) Scatter plot between array N1 and array N3. (*C*) Scatter plot between array N2 and array N3. Diagonal lines represent 2x, 3x, 5x and 10x difference between signal detection values. Red dots = P-P, present in both experiments. Black dots = P-A, present in the first experiment and absent in the second. Green dots = A-P, absent in the first experiment and present in the second. Yellow dots = A-A, A-M, M-A, M-M, absent or marginal in the first and/or second experiment. Blue dots = M-P, P-M, present or marginal in the first or second experiment.

We performed microarray hybridization for three samples of amplified periglomerular cell RNA and analyzed gene expression based on the signal detection value (Table 1). Full analysis of microarray data as well as raw data can be found on http://www.ebi.ac.uk/miamexpress (accession number is E-MEXP-1234, public access is available from the 10.11.2007). Since the background signal detection value is about 50–70, genes with a signal detection value higher than 100 were further analyzed using GeneOntology (GO) and KEGG Pathway databases. The reproducibility and quality of the data were demonstrated by scatter plot analysis (Fig. 3).

**Table 1:** Microarray and qRT-PCR analysis for expressed and non-expressed genes in periglomerular cells^1^

Gene	Signal detection value	C_t_ 2
*Actb*	18925	17.72
*Egfp*	-3	18.17
*Htr3a*	607	21.12
*Dcx*	11202	22.80
*Gad1*	28449	19.50
*Mog*	<100	29.53
*Omp*	<100	30.78
*Csf1*	161	30.09
*Syn1*	269	32.02
*Gria3*	277	28.21

1 sequences of primers used for qRT-PCR are shown in Table 2.

2 C_t_–cycle of detection of amplifying PCR product.

3 Egfp gene cannot be detected by Affymetrix Gene Chip Mouse Genome Array.

Subsequently, we confirmed microarray expression data by quantitative real-time PCR (qRT-PCR) (Table 1). This analysis included both genes whose expression in periglomerular cells had been shown as well as genes whose lack of expression had been documented. As expected, among prominently expressed mRNAs were those encoding EGFP and the 5HT3_A_ receptor, in accordance with the green fluorescent cells expressing EGFP from the 5HT3_A_ gene (*Htr3a*) promoter. The expression of 5HT3_A_ gene was somewhat under-estimated by the microarray data. This is most likely due to the fact that the probe is located further 5′ compared to the qRT-PCR primers. Since material from two rounds of amplification was used, differential detectability by the two approaches can be expected. The migratory neuroblast and GABAergic phenotype of periglomerular cells was reflected in the expression of *Dcx* [14] (immature neurons) and *Gad1* [15] (GAD67 protein in GABAergic interneurons), respectively. Conversely, transcription levels of genes specific for neighboring regions or cell types such as oligodendrocytes (*Mog* [16]), olfactory sensory cells (*Omp* [17]), mitral cells (*Csf1* [18]), external plexiform layer (*Gria3*, GluR3 protein [19]) and mature neurons (*Syn1* [20]) were low.

Estimating that β-actin mRNA occurs in 500–2,000 copies per cell, we suggest that these negative markers are represented in our samples by at most 1 copy for every 10 cells, because their amplification required 11–18 cycles more than β-actin mRNA. Hence, it can be inferred that there was only a small percentage of non-periglomerular cells among the 3,000–5,000 dissected cells, which demonstrates the remarkable sensitivity of our technique.

## Discussion

**Figure 4:**
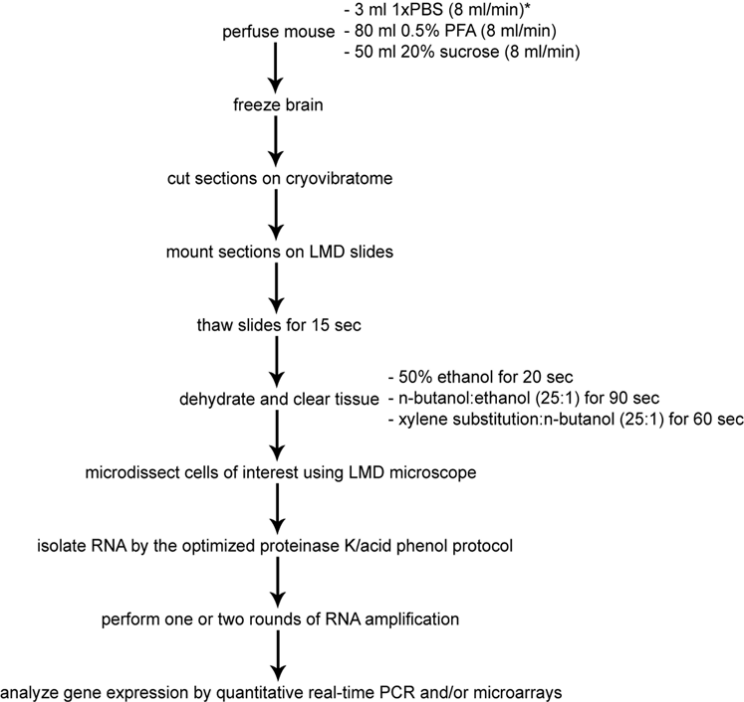
Flow diagram of the whole technique described. Asterisk–perfusion conditions for P15 mouse.

We developed a technique of high quality RNA isolation from *in vivo* fluorescent cells that is useful for gene expression pattern analysis of defined cell populations (Fig. 4). We applied this technique for a specific interneuronal cell type–periglomerular neurons–of mouse brain, the most complex mouse tissue, employing a transgenic mouse line expressing EGFP from the promoter for the serotonergic receptor 5HT3_A_. Using this technique we obtained periglomerular cell RNA of excellent quality and confirmed its cell type specificity. We amplified isolated RNA and showed that it could be used in gene expression studies with qRT-PCR and/or microarrays.

To date there have been only few attempts to separate *in vivo* fluorescent cells from complex tissue using LMD. One reported approach is based on alcohol fixed slices that enabled the identification and microdissection of fluorescent cells [21]. However, the success of these experiments required the additional generation of transgenic mice expressing the nuclear version of the fluorescent protein. This was necessary in order to preserve the fluorescence in the cells and to avoid leaching and diffusion of the fluorescent marker. Thus, although it was possible in the study to obtain specific and high quality mRNA from fluorescent cells, this approach only rarely will be applicable since in most transgenic mouse lines generated so far the cytosolic form of the fluorescent protein has been used. In other report authors used 4% PFA to preserve fluorescence [22]. Although they were able to fix fluorescence in the cells, they did not determine whether the quality and yield of RNA isolated from the fixed tissue was appropriate for gene expression analysis. As reported by other labs [23]–[27], and in our hands too, it was not possible to obtain RNA of good quality from fixed tissue using the conventional guanidine isothiocyanate method or the standard proteinase K method. The main problems concerning RNA isolation from aldehyde-fixed tissues are RNA modifications and RNA trapping [12]. RNA modifications could be resolved by SDS and acid phenol treatment [12], [28], which revert methylene crosslinks formed by paraformaldehyde. Furthermore, any hydration of methylene bridges in RNA-protein complexes will set RNA free due to the preference of SDS to bind to the protein part of methylene crosslinks and the repulsive character of the bulky SDS-protein compound [12]. It is very important to use acid phenol, as in addition to reversing crosslinks, it also effectively deprotonates methylene links due to low pH. We also overcame the problem of RNA trapping. Aldehyde fixatives induce the formation of extensive inter- and intra-molecular networks that result in RNA trapping and low RNA yield, especially for high molecular weight RNAs. As a result, RNA of shorter size would be isolated preferentially over longer RNA, which would compromise the subsequent gene expression analysis. This problem was resolved using excessive concentrations of proteinase K and SDS in the lysis solution and an optimized buffer for proteinase K digestion. We also optimized the lysis solution for RNase inactivation.

Using an optimized proteinase K/acid phenol procedure for RNA isolation, we obtained RNA from fixed tissue with the quality of unfixed frozen tissue RNA and having a 28S∶18S rRNA ratio of ∼2∶1. We showed that the RNA amplification profile of RNA isolated by our method resembled that of Ambion Control RNA, and hence, the representation of different sequence species in the mRNA population appears not to change during amplification. We were able to obtain sufficient RNA for several microarray hybridizations. Comparing three independent experiments, we provide evidence for excellent reproducibility and hence the reliability of the microarray data (Fig. 3, full analysis data can be found on http://www.ebi.ac.uk/miamexpress, accession number is E-MEXP-1234, public access is available from the 10.11.2007). Furthermore, we demonstrated the specificity of the amplified RNA pool by analyzing expression levels of different positive and negative gene markers using microarray hybridization and qRT-PCR. Positive markers were genes known to be expressed at high levels in periglomerular cells [14], whereas negative markers were genes that were shown to be expressed in surrounding cell populations but not in the cell type analyzed here. Thus, we validated both usefulness and specificity of our technique for global gene expression analysis of *in vivo* fluorescent neural cells

Fluorescent Activated Cell Sorting (FACS) appears to be a viable alternative, as it allows the isolation of small subpopulations of *in vivo* fluorescent cells with excellent specificity [29]. However, during the pre-sorting and sorting steps, cells are incubated in a non-natural microenvironment for several hours. Moreover, sorting requires tissue homogenization by proteases for a prolonged time (e. g. 45 min incubation at 37°C to homogenize striata [30]). All these steps could dramatically change gene expression patterns, especially during development, when cells undergo rapid changes in gene expression. The technique described here employs rapid tissue fixation by PFA and hence, gene expression remain unchanged. Furthermore, this approach guarantees a better cell resolution than that obtained by FACS, which is an important consideration, especially, when separating fluorescent cell populations that are located in close proximity but may have different functions and hence different gene expression.

Indeed, our technique should prove of considerable advantage for the analysis of gene expression of specific cell populations in the many existing transgenic mouse lines having genes tagged by fluorescent protein expression. For example, about 450 transgenic mouse lines expressing EGFP from specific promoters have been generated (80 new lines are under investigation [8]) by the Gene Expression Nervous System ATlas Program (GENSAT) to date and by several other research labs (e.g. [9]). In addition to transgenic animals in which a fluorescent protein is expressed from a specific promoter, other methods exist for fluorescence delivery to specific cell types/populations. The most prominent one is viral delivery of a specific construct, which is expressed only in particular cells [11]. Others include naked DNA delivery [10] as well as specific protein labeling [31]. Such a diversity of *in vivo* fluorescent labeling of specific cells makes the technique described here both useful and promising for the analysis of gene expression pattern of distinct cell types/populations.

## Materials and Methods

### Brain fixation for Laser Microdissection (LMD)

Transgenic 5HT3_A_-EGFP mice (P15) received an intraperitoneal injection of anesthesia (ketanest 18 mg/ml, xylasin 0.24%, final concentration) and were transcardially perfused by 1xPBS for 20 sec (8 ml/min), 0.5% or 2% paraformaldehyde (PFA) for 10 min (8 ml/min) and then by 20% sucrose for 7 min (8 ml/min). After perfusion, the brain was rapidly removed from the skull and frozen on dry ice. Brains were stored at −80°C.

### Brain fixation-standard protocol

Transgenic 5HT3_A_-EGFP mice (P15) received an intraperitoneal injection of anesthesia (ketanest 18 mg/ml, xylasin 0.24%, final concentration) and were transcardially perfused by 1xPBS for 20 sec (8 ml/min) and 4% PFA for 20 min (8 ml/min). After perfusion, the brains were fixed in 4% PFA overnight and then washed by 1xPBS. Brains were stored at 4°C.

### Preparation of sections to visualize *in vivo* fluorescence using the standard protocol

75 µm-thick sagittal brain sections were made from transgenic 5HT3_A_-EGFP mice (P15) using a vibratome (Leica VT1000S). Sections were mounted onto slides with Moviol (Roth, Germany) and were subsequently analyzed using an upright fluorescent microscope (Zeiss Axioplan 2).

### Preparation of sections for LMD

Frozen brains were embedded in Tissue Freezing Medium (Leica Instruments, Germany) at −20°C, and 6–8 µm-thick sagittal brain sections were made using the vibratome Microm HM500 (MICROM International, Germany). Sections were mounted on membrane polyester slides (Leica Microsystems, Germany), briefly thawed and dehydrated by sequential incubation in 50% ethanol for 20 sec and n-butanol:ethanol (25∶1) for 90 sec, followed by 60 sec of xylene substitution (Sigma-Aldrich, Germany) clearing, to which 1/25 volume of n-butanol was added. Sections were dried for 5 min and used for LMD.

### Laser Microdissection

LMD was performed on a Leica LMD6000 B microscope (Leica Microsystems, Germany). Approximately 3,000–5,000 cells were dissected from 50–70 sagittal brain sections of 6–8 µm from one transgenic 5HT3_A_-EGFP mouse within 4–5 hr. Cells were dissected into dry 0.2 ml tube caps coated with silicon (Leica Microsystems, Germany) at the power 55–57 and speed 3 using a 40x objective or at power 53–54 and speed 3 using a 63x objective. To facilitate collection of such a high cell number, ensembles of adjacent fluorescent cells were often co-dissected during the same laser movement. Three mice were used to assess reproducibility.

### RNA isolation

#### Optimized proteinase K/acid phenol method

Directly following microdissection, the collected 3,000–5,000 cells were lysed in 100 µl of lysis solution [10 mM Tris-HCl (pH 7.9), 50 mM EDTA (pH 7.9), 0.2 M NaCl, 2.2% SDS, 0.5 µg/µl AntiRNase (Ambion, USA) and 1000 µg/ml proteinase K (Ambion, USA)] at 55°C for 3h with vigorous shaking. The volume was adjusted to 600 µl by water followed by adding an equal volume of phenol, pH 4.2. The solution was vigorously mixed during 2 min, left for 5 min on ice and centrifuged at 14,000 g for 10 min at 4°C. The aqueous phase was aspirated into a fresh tube and subjected to equal volume of phenol∶chloroform (1∶1) treatment. The mixture was vortexed during 2 min, left for 5 min and centrifuged at 14,000 g for 10 min at 4°C. Again, the aqueous phase was transferred into a fresh tube and mixed with an equal volume of isopropanol and 20 µg of glycogen (Ambion, USA). The mixture was incubated at –20°C for 30 min and centrifuged at 14,000 g for 10 min at 4°C. The pellet was washed with 600 µl of cold 70% ethanol, air-dried and dissolved in 26 µl of water and 3 µl of 10x DNase buffer (Ambion, USA). To eliminate any remaining DNA, the mixture was treated by 1 U of DNase I (Ambion, USA) for 15 min at 37°C and purified by use of RNeasy MinElute Cleanup Kit (QIAGEN, Germany) according to manufacturer's recommendations, except for using RPE solution (included in the Cleanup Kit) instead of 80% ethanol in the second washing step and performing the elution step twice, each with 15 µl of water. Obtained RNA (typically 6–9 ng) was concentrated by Eppendorf Concentrator 5301 (Eppendorf, Germany) and analyzed by Bioanalyzer 2100 (Agilent, USA).

#### Conventional methods

RNA was isolated either by the TRIzol (Invitrogen, Germany) method or by use of an RNeasy Micro Kit (QIAGEN, Germany) according to manufacturers' recommendations.

### RNA amplification

Total RNA (2–3 ng) was amplified using the MessageAmp II aRNA Amplification Kit (Ambion, USA) according to manufacturer's recommendations. During the T7 *in vitro* transcription step, the mixture was incubated at 37°C for 14–16h. After each amplification round, the RNA was analyzed by Bioanalyzer 2100 (Agilent, USA). We typically obtained 100–200 ng and 50–100 µg of amplified RNA after the first and second amplification round, respectively. Amplifications were done from three periglomerular cell RNA samples obtained from three 5HT3_A_-EGFP mice. For microarray hybridization, a second round RNA amplification was performed using biotinylated nucleotides.

### Microarray hybridization

Microarray hybridization was performed according to the Affymetrix GeneChip Expression Analysis Technical Manual (www.affymetrix.com). For 3 samples of periglomerular cell RNA, 10 µg of biotinylated cRNA was hybridized onto an Affymetrix Gene Chip Mouse Genome 430A 2.0 arrays. Arrays were scanned with the Affymetrix GeneArray 2500 scanner. Gene expression data were obtained using the Affymetrix software.

### Microarray data analysis

Microarray data analysis was carried out using R packages gcrma, affy and genefilter of Bioconducter project [32]. The genes that had 100% P-calls and signal detection value of more than 100 in all three arrays were filtered out from the raw hybridization data.

### cDNA synthesis and quantitative real-time PCR (qRT-PCR)

cDNA was synthesized from 2 µg of amplified RNA by High Capacity cDNA Reverse Transcription Kit (Applied Biosystems, Germany). QRT-PCR was performed on a TaqMan ABI Prism 7000 Sequence detection system (Applied Biosystems, Germany) using Power SYBR Green PCR Master Mix (Applied Biosystems, Germany) with the following parameters: 50°C–2 min, 95°C–10 min followed by 45 cycles of 95°C–15 sec and 60°C–1 min. Sequences of primers for real-time PCR are listed in the Table 2. MRNA levels detected by qRT-PCR were normalized to mRNA levels for β-actin, a well-known house-keeping gene. Quantifications were made using the relative standard curve method with comparison to Stratagene QPCR Mouse total RNA (Stratagene Europe, Netherlands). To create the standard curves, a dilution series across 5 orders of magnitude of cDNA concentration, generated from Stratagene QPCR Mouse total RNA, was prepared for each experiment. Each sample was amplified in triplicate.

**Table 2:** Primers sequences used for qRT-PCR analysis

Gene	Primers used
*Actb*	CTGGAACGGTGAAGGCGACA
	GGTGAGGGACTTCCTGTAACCACT
*Egfp*	CCACTACCTGAGCACCCAGTC
	CACGAACTCCAGCAGGACCA
*Htr3a*	ACTCAGTCTTCCTCATCATCGTGTCAG
	TGGTCTCAGCGAGGCTTATCACT
*Dcx*	ACTTGTGAGGCATTTGGAGACATCAGA
	CCTTACCTTTGCTGACTGGAGCCTA
*Gad1*	CCTCAGGCTGTATGTCAGATGTTCTCAA
	GCTAAGCGAGTCACAGAGATTGGTCAT
*Mog*	CAACTGGCTGCACCGAAGACT
	CGCTCCAGGAAGACACAACCAT
*Omp*	GAGGCAGGAGATAGGCTGTGGTA
	CGGCAAGCATGTTATGGAGCAGA
*Csf1*	GCCAGGCTTGTCTGTGGTGA
	TAGCCAGGGAGGGCAGGAA
*Syn1*	CCCAGCCAGGATGTGCCA
	AGGCATTGGTCAGAGACTGGGATT
*Gria3*	GTCATCAGACCAGCCAGAGGAAATAGT
	CCAATGCACGTTACTGATGAGAGCATAC
